# PqsE adapts the activity of the *Pseudomonas aeruginosa* quorum-sensing transcription factor RhlR to both autoinducer concentration and promoter sequence identity

**DOI:** 10.1128/jb.00516-24

**Published:** 2025-04-17

**Authors:** Bilalay V. Tchadi, Jesse J. Derringer, Anna K. Detweiler, Isabelle R. Taylor

**Affiliations:** 1 Department of Chemistry, William & Mary 427305 , Williamsburg, Virginia, USA; University of California San Francisco, San Francisco, California, USA

**Keywords:** *Pseudomonas aeruginosa*, quorum sensing, gene regulation, protein-protein interaction

## Abstract

*Pseudomonas aeruginosa* is an opportunistic human pathogen that poses a significant health threat. Many pathogenic behaviors of *P. aeruginosa* are under control of the bacterial cell-cell communication system known as quorum sensing (QS). One of the QS master regulators, RhlR, is a receptor/transcription factor that not only relies on binding of its canonical ligand, *N*-butyrylhomoserine lactone (C4-HSL), but additionally requires a protein-protein interaction with the enzyme, PqsE. We constructed heterologous reporter strains in *Escherichia coli* that allow measurements of the reliance of RhlR on C4-HSL and/or PqsE binding for the ability to activate transcription of three RhlR-regulated genes: *rhlA* (PqsE independent), *phzM* (PqsE dependent), and *azeB* (PqsE inhibited). Analogous assays measuring activation of the three genes in *P. aeruginosa* were performed, and the patterns observed correlated tightly with the heterologous reporter assays. These results confirm that the binding of PqsE to RhlR is able to fine-tune RhlR transcription factor activity in a promoter-specific manner and prove that this ability is independent of other factors present in *P. aeruginosa*.

**IMPORTANCE:**

*Pseudomonas aeruginosa* is an opportunistic human pathogen that can cause fatal infections. There exists an urgent need for new, effective antimicrobial agents to combat *P. aeruginosa*. The PqsE-RhlR protein-protein interaction is essential for *P. aeruginosa* to be able to make toxins, form biofilms, and infect host organisms. In this study, we use both non-native models in *Escherichia coli* and measurements of gene expression/toxin production in *P. aeruginosa* to show that the PqsE-RhlR interaction enables fine-tuned gene expression and a heightened ability of *P. aeruginosa* to adapt to external conditions. These findings will be highly valuable as continued efforts are made to design inhibitors of the PqsE-RhlR interaction and test them as potential antimicrobial agents against *P. aeruginosa* infections.

## INTRODUCTION

The opportunistic pathogen, *Pseudomonas aeruginosa*, is a gram-negative bacterium that is responsible for causing life-threatening infections in immunocompromised individuals. Due to its heightened ability to resist antibiotics, there is a severe lack of treatment options for those who acquire *P. aeruginosa* infections (1, 2). Resistance to antibiotic treatments as well as several of the virulence phenotypes exhibited by *P. aeruginosa*, such as toxin production and biofilm formation, is under control of the bacterial cell-cell communication process, called quorum sensing (QS) (3–6). QS is the process by which bacteria produce, release, and detect small molecule signals (autoinducers) in order to orchestrate coordinated, cell density-dependent group behaviors (3). When the extracellular concentration of autoinducers reaches a particular threshold, bacteria make a coordinated lifestyle switch, activating the transcription of genes that promote group behaviors. When *P. aeruginosa* reaches high cell density, genes responsible for producing virulence factors, such as pyocyanin and rhamnolipids, become activated by the QS receptor/transcription factors (7).

**Fig 1:**
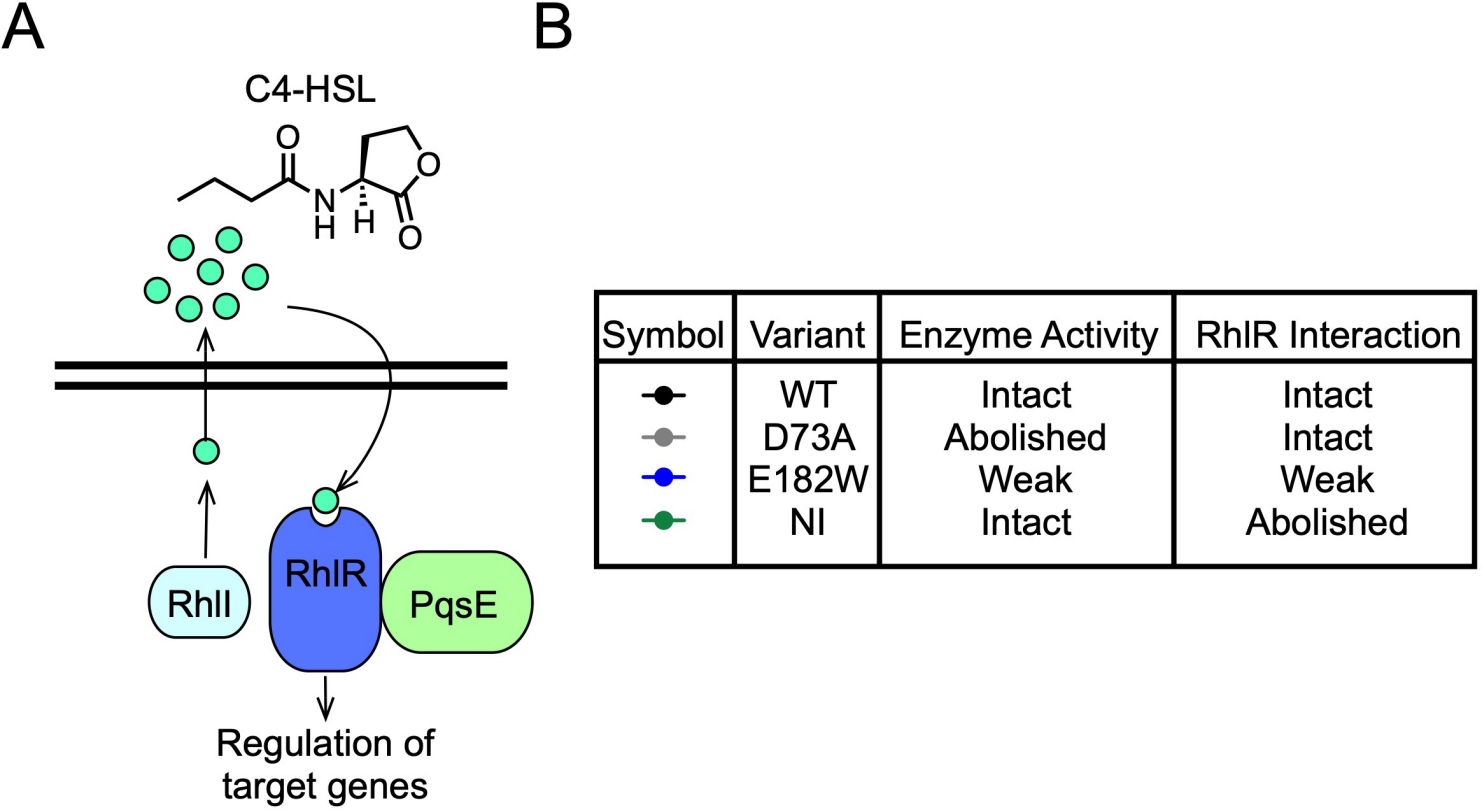
RhlR activity is influenced by two binding partners, C4-HSL and PqsE. (**A**) The Rhl branch of QS in *P. aeruginosa*. See text for details. (**B**) PqsE variants used in this study. Each variant has been previously characterized for its enzymatic capability and the strength of its interaction with RhlR.

*P. aeruginosa* has multiple QS circuits in place that regulate a complex network of genes. There are two LuxI/R-type autoinducer synthase/receptor pairs: LasI and LasR, which are responsible for the synthesis and detection of *N*-(3-oxododecanoyl)homoserine lactone (3-oxo-C12-HSL), respectively, and RhlI and RhlR, responsible for the synthesis and detection of *N*-butyrylhomoserine lactone (C4-HSL), respectively (Fig. 1A) (8, 9). Although *rhlI* expression is under the control of LasR, suggesting that LasR is at the top of the QS regulatory hierarchy, mounting evidence has shown that intact signaling through the Las branch is not required for infection. Rather, mutations in *las* often produce hypervirulent *P. aeruginosa* strains (10, 11). Contrary to this, *rhlR* mutants rarely occur in clinical isolates of *P. aeruginosa* (12). It has recently been suggested that understanding the QS hierarchy be “rewritten,” placing RhlR at the top as the master regulator of quorum-sensing phenotypes in *P. aeruginosa* (13).

In addition to a newfound appreciation for the importance of RhlR regulatory function in *P. aeruginosa* virulence, recent progress has been made in understanding how RhlR function differs from that of other LuxR family receptors. In particular, RhlR requires a protein-protein interaction with a metallo-β-hydrolase from a separate QS system, PqsE (14, 15). The interaction between PqsE and RhlR is completely independent from the enzymatic activity of PqsE (14, 16). On the other hand, when mutations are introduced to either *rhlR* or *pqsE* that disrupt the formation of the PqsE:RhlR complex, *P. aeruginosa* can no longer produce pyocyanin or successfully colonize the lungs of a mouse (17). It was shown that by forming a complex with RhlR, PqsE enhances the affinity of RhlR for binding some of its target promoter sequences (15). Furthermore, recent structural analyses have illustrated that the two proteins form a functional tetrameric complex, consisting of a PqsE dimer and an RhlR dimer (18, 19). These recent findings are consistent with the longstanding observation that, unlike other LuxR family receptors, binding of RhlR to its native ligand, C4-HSL, is not sufficient to solubilize the protein when expressed recombinantly in *Escherichia coli* (20). This complication had prevented structural characterization of RhlR prior to the discovery of the PqsE-RhlR protein-protein interaction.

The reliance on a protein-protein interaction for full functionality is a feature that, to date, has only been observed for RhlR of the LuxR family of transcription factors. Recent efforts have focused on understanding what expanded or nuanced activity the interaction with PqsE bestows upon RhlR. A transcriptional analysis revealed RhlR regulates different subsets of genes with varying dependencies on the interaction with PqsE (21). Furthermore, a recent chromatin immunoprecipitation and sequencing (ChIP-seq) analysis identified the classes of genes in the RhlR regulon that were dependent on C4-HSL binding, PqsE binding, both, or neither (22). The study identified 40 DNA-binding sites for RhlR, and the genes associated with these binding sites encoded a variety of proteins largely involved in the production of toxins and virulence factors. Another recent study suggested that PqsE allows for condition-dependent gene regulation (23). These recent studies highlight an important feature of RhlR transcription factor activity in that it can be fine-tuned or adjusted by two independent binding partners: one a protein (PqsE) and the other a small molecule ligand (C4-HSL) (Fig. 1A).

We set out to achieve two goals: (1) characterize regulation of genes representing different classes of RhlR-controlled targets using a heterologous *E. coli* bioluminescent reporter and (2) determine how well the heterologous reporter recapitulates regulation of these genes in *P. aeruginosa*. The *E. coli* system allows for the isolation of PqsE:RhlR:C4-HSL function from other potential regulatory factors present in *P. aeruginosa* and thus allows for the study of the degree to which C4-HSL and/or PqsE directly determine the functionality of RhlR in a promoter-specific manner. The target gene promoter sequences chosen were those for *rhlA*, the canonical target gene of RhlR responsible for the production of rhamnolipids (24), *phzM*, a gene in the phenazine biosynthetic pathway that is necessary for the production of pyocyanin (25), and *azeB*, a gene encoding a non-ribosomal peptide synthetase that is part of a newly characterized biosynthetic gene cluster responsible for the production of various azetidomonamide and diazetidomonapyridone derivatives with unknown function (26, 27). These three genes represent different classes of RhlR-regulated genes defined by the previous ChIP-seq analysis, with both *rhlA* and *azeB* demonstrating PqsE independence, while *phzM* depends on both PqsE and C4-HSL (22). One intriguing observation from the ChIP-seq as well as previous transcriptomics analyses (21) was that the dependence on either C4-HSL or PqsE was not binary: rather dependence on each binding partner existed on a spectrum with some genes being more or less dependent on C4-HSL (assessed by comparing WT *P. aeruginosa* to the ∆*rhlI* mutant, incapable of producing C4-HSL) or PqsE (assessed by comparing WT *P. aeruginosa* to the ∆*pqsE* mutant). To further investigate this pattern, we probed RhlR transcriptional activity for each specific target promoter in the presence of varying C4-HSL concentrations, as well as when co-expressed with variants of PqsE that have different affinities for RhlR. The PqsE(R243A/R246A/R247A), from now on referred to as PqsE(NI) for “non-interacting,” variant is completely incapable of self-dimerizing to interact with RhlR, and the PqsE(E182W) variant has a severely weakened interaction with RhlR, which can be circumvented by the addition of high concentrations of C4-HSL to restore virulence factor production in strains harboring this mutation. Finally, the PqsE(D73A) variant possesses a similar affinity for RhlR to that of PqsE(WT) but is catalytically inactive (Fig. 1B). This panel of PqsE mutants as well as the exogenous addition of C4-HSL allowed for the precise measurement of dependence on PqsE vs C4-HSL for full functionality of RhlR.

## RESULTS

### Transcription of *rhlA* is enhanced by, but not dependent on, PqsE binding to RhlR

We were initially interested in characterizing the efficiency of RhlR-activated transcription of its canonical target gene, *rhlA*, when exposed to a range of concentrations of C4-HSL and either in a complex with PqsE or alone. *rhlA* encodes one of the genes necessary for the production of a class of biosurfactants called rhamnolipids. Rhamnolipids play roles in virulence, biofilm formation, and swarming motility (24, 28, 29). Previous characterization of RhlR promoter occupancy identified that the binding of RhlR to the *rhlA* promoter was possible in the ∆*pqsE* mutant but not observed in the ∆*rhlI* mutant (22). This suggests that RhlR can activate the transcription of *rhlA* independently of PqsE but requires the binding of C4-HSL to do so.

**Fig 2:**
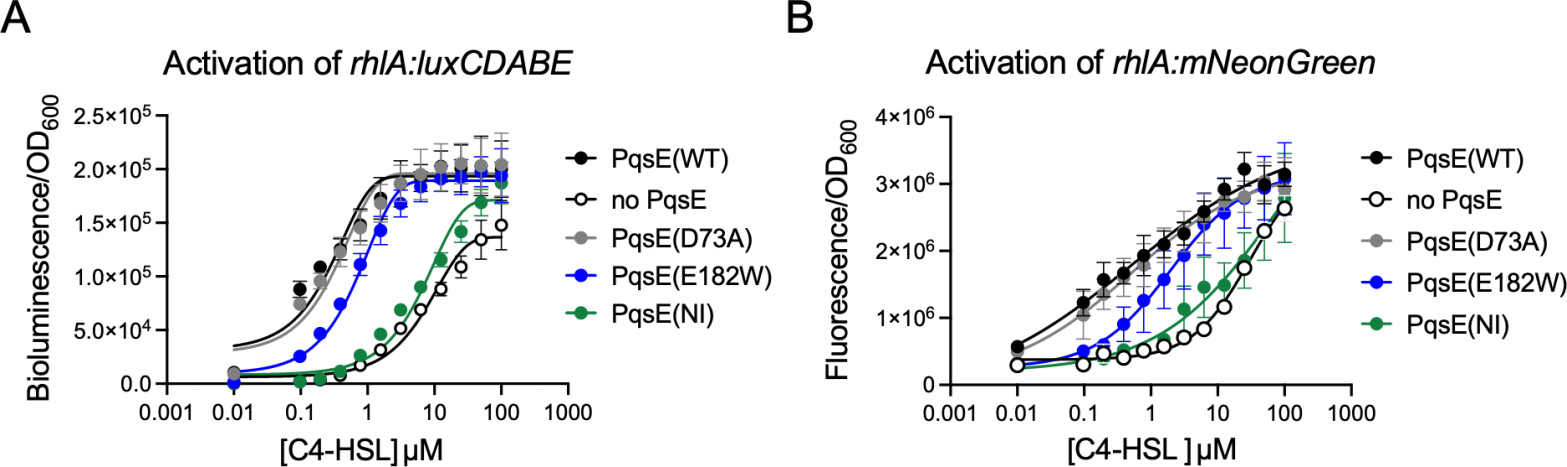
Activation of *rhlA* by RhlR-C4-HSL is enhanced by, but not reliant on, PqsE binding. (**A**) Activation of *rhlA-lux* in *E. coli* is reported as luminescence divided by OD_600_, and the measurements shown were collected after 17 h of growth. (**B**) Activation of *rhlA:mNeongreen* in *P. aeruginosa* is reported as fluorescence (excitation: 485 nm, emission: 535 nm) divided by OD_600_, and the measurements shown were collected after 17 h of growth. Stimulation curves were fit to data collected from two independent experiments performed in duplicate. Error bars represent standard deviations.

Using a previously reported heterologous *E. coli* system to measure the transcriptional activation of *rhlA:luxCDABE* via bioluminescence, we determined the sensitivity of RhlR to exogenously added C4-HSL in the presence or absence of an interaction with PqsE (Fig. 2A). When PqsE was not co-expressed with RhlR in the heterologous system (this strain harbored an empty pACYC184 plasmid with no *pqsE* allele encoded on it), RhlR-activated transcription of *rhlA:luxCDABE* could be fit with a sigmoidal stimulation curve with an EC_50_ of approximately 12 µM (11.8 ± 8 µM) C4-HSL. By comparison, the co-expression of PqsE(WT) led to a 100-fold increase in sensitivity of RhlR to C4-HSL (EC_50_ = 118 ± 73 nM). These results confirm those previously reported from experiments using an *E. coli rhlA-lacZ* reporter (30). Three additional PqsE variants were tested in this system, all of which have been previously characterized for their apparent affinity for RhlR (17). Briefly, PqsE(D73A) has approximately the same affinity for RhlR as PqsE(WT). PqsE(E182W) is an inhibitor mimetic mutant that has a severely weakened affinity for RhlR and is incapable of driving virulence phenotypes when expressed in *P. aeruginosa*. Finally, PqsE(NI) is completely incapable of forming a complex with RhlR. When tested in the heterologous model for activation of *rhlA:luxCDABE* transcription, the patterns observed for each mutant closely reflected their varying abilities to form a complex with RhlR. Expression of PqsE(D73A) gave a result that closely resembled that of PqsE(WT) (EC_50_ = 205 ± 23 nM), while expression of PqsE(NI) closely resembled that of no PqsE (EC_50_ = 10.4 ± 4 µM). Finally, expression of the PqsE(E182W) mutant, which has a weak affinity for RhlR, produced an EC_50_ of approximately 600 nM (614 ± 2 nM), reflecting an intermediate level of RhlR sensitivity to C4-HSL.

An analogous experiment was performed in a *∆rhlI ∆pqsE P. aeruginosa* PA14 strain harboring a *rhlA:mNeonGreen* reporter (Fig. 2B). Strains expressing each of the *pqsE* alleles tested above on the pUCP18 plasmid were generated in this *rhlA* reporter background in order to measure the dependence of RhlR on C4-HSL binding vs PqsE binding for activating transcription of *rhlA* in *P. aeruginosa*. The resulting C4-HSL stimulation curves closely resembled those generated using the *E. coli* heterologous reporter, albeit with slightly altered concentration ranges. Both the PqsE(WT) and PqsE(D73A) strains exhibited mid-nanomolar EC_50_ values (516 ± 25 and 498 ± 455 nM, respectively), and the strains without an intact PqsE-RhlR interaction (the no PqsE and PqsE(NI) strains) were approximately 100 times less sensitive to C4-HSL (EC_50_ values of 31.1 ± 4 and 71.9 ± 36 µM, respectively). As in the *E. coli* reporter, the *P. aeruginosa* reporter strain harboring the PqsE(E182W) variant displayed an intermediate phenotype, with an EC_50_ of 2.0 ± 1 µM. The alignment of results in the heterologous reporter with those obtained in *P. aeruginosa* suggests that there are no additional factors present in the native bacterium that influence RhlR transcriptional control over *rhlA* expression other than C4-HSL and PqsE.

### Transcription of *phzM* is dependent on PqsE binding to RhlR and inhibited at high C4-HSL concentrations

The original characterization of the PqsE-RhlR interaction revealed that this interaction was necessary for the ability of *P. aeruginosa* to produce the toxin, pyocyanin. *P. aeruginosa* secretes pyocyanin as a defense mechanism against host or other bacterial cells, and production of the toxin has been correlated to the ability of *P. aeruginosa* to stage an infection (31, 32). Pyocyanin is a member of a class of molecules called phenazines, produced from the action of the *phzABCDEFG* operon along with *phzM* and *phzS* (25). All of the *phz* genes necessary for pyocyanin production were identified to have strong dependence on both C4-HSL and PqsE for binding of RhlR to their respective promoter regions and transcription (21, 22, 33, 34). Notably, *phzM* shares the same promoter region with the *phzA-G1* operon but is oriented on the opposite strand from the operon. Therefore, it was particularly of interest to determine whether RhlR does, in fact, activate *phzM* expression directly.

**Fig 3:**
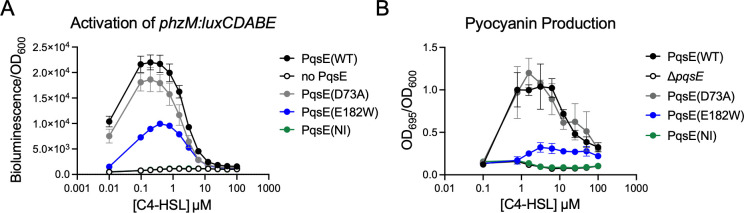
Activation of *phzM* by RhlR-C4-HSL is dependent on PqsE binding. (**A**) Activation of *phzM-lux* in *E. coli* is reported as luminescence divided by OD_600_, and the measurements shown were collected after 17 h of growth. Results shown are the average of two independent experiments performed in duplicate. (**B**) Pyocyanin production was measured as OD_695_ of the cell-free supernatants and normalized to OD_600_ of the resuspended cell pellets. Measurements were taken after 17 h of growth. Results shown are the average of two biological replicates performed in duplicate. Error bars represent standard deviations.

We adapted the heterologous reporter system to feature the promoter region for the gene, *phzM*, fused to the *luxCDABE* operon and used this reporter to measure the activity of RhlR at a range of C4-HSL concentrations and in the presence or absence of the PqsE interaction (Fig. 3A). In contrast to the *rhlA* reporter, no increase in bioluminescence was observed at any concentration of C4-HSL when PqsE was not co-expressed with RhlR. In the presence of PqsE(WT), RhlR was able to activate transcription, producing peak luminescence at approximately 200 nM C4-HSL. Interestingly, at concentrations above ~500 nM C4-HSL, luminescence began to decrease, falling back down to baseline levels at ~10 µM C4-HSL. Like with the *rhlA* reporter, the PqsE variants followed a trend that reflects the strength of the interaction with RhlR. The PqsE(D73A) and PqsE(NI) mutants resembled PqsE(WT) and no PqsE, respectively, and expression of the PqsE(E182W) mutant produced an intermediate phenotype.

The enzyme, PhzM, catalyzes the methylation of phenazine-1-carboxylic acid, a necessary step in the production of pyocyanin as opposed to other phenazine precursors. Therefore, in order to determine how well results from the heterologous reporter replicated transcriptional regulation of *phzM* in *P. aeruginosa*, we measured pyocyanin production in ∆*rhlI P. aeruginosa* strains expressing the panel of *pqsE* variants at their native locus with exogenous addition of C4-HSL at a range of concentrations (Fig. 3B). As in the heterologous reporter, the strains that lacked an intact PqsE-RhlR interaction (no PqsE and PqsE(NI)) did not produce pyocyanin at any concentration of C4-HSL. Also mirroring the heterologous reporter, both PqsE(WT) and PqsE(D73A) were able to produce pyocyanin, with peak production observed at approximately 1 µM C4-HSL. As with the heterologous reporter, pyocyanin production decreased to near baseline levels at higher concentrations of C4-HSL, consistent with previous observations (35). And finally, the PqsE(E182W) mutant, which has a weakened affinity for RhlR, displayed an intermediate phenotype, where peak pyocyanin production amounted to 26% of that produced by the strain expressing PqsE(WT). This result correlates well with previous studies in which PqsE(E182W)-expressing strains exhibited a similar impairment in pyocyanin production when *rhlI* was not deleted (14). We note that pyocyanin is the end biosynthetic product of enzymes encoded by multiple genes in addition to *phzM*, and the measurement of pyocyanin production could reflect regulation of the *phzA-G* operons as well as *phzS*. It would also be of interest to measure the production of precursors to pyocyanin along with testing heterologous reporter systems measuring *phzA1*, *phzA2*, and *phzS* activation. Our group is currently constructing heterologous reporter systems to study the activation of *phzA1*, *phzA2*, and *phzS* by the PqsE:RhlR:C4-HSL system.

### Transcription of *azeB* is stimulated by PqsE binding in the absence of C4-HSL but inhibited by binding of PqsE to RhlR:C4-HSL

The *aze* operon consists of 10 genes encoding enzymes that collectively produce a series of azetidine-containing natural products, including the molecule azabicyclene. This gene cluster is highly conserved among *P. aeruginosa* strains, with further conservation among 12 additional *Pseudomonas* species (26). While the precise roles of these natural products are unknown, the deletion of certain genes in the cluster increased the ability of *P. aeruginosa* PAO1 to colonize the mouse lung (36). Interestingly, the entire *aze* operon, along with *azeA*, has been identified as being part of the core RhlR regulon in clinical isolates of *P. aeruginosa* harboring mutations in the *las* system (33). Furthermore, the binding of RhlR to the *azeB* promoter and transcription was shown to be independent of PqsE (21, 22). For all of the above reasons, regulation of the *az*e gene cluster by PqsE:RhlR was of great interest. We therefore constructed luminescent reporters to study the transcription of *azeB* in both *E. coli* and *P. aeruginosa*.

**Fig 4:**
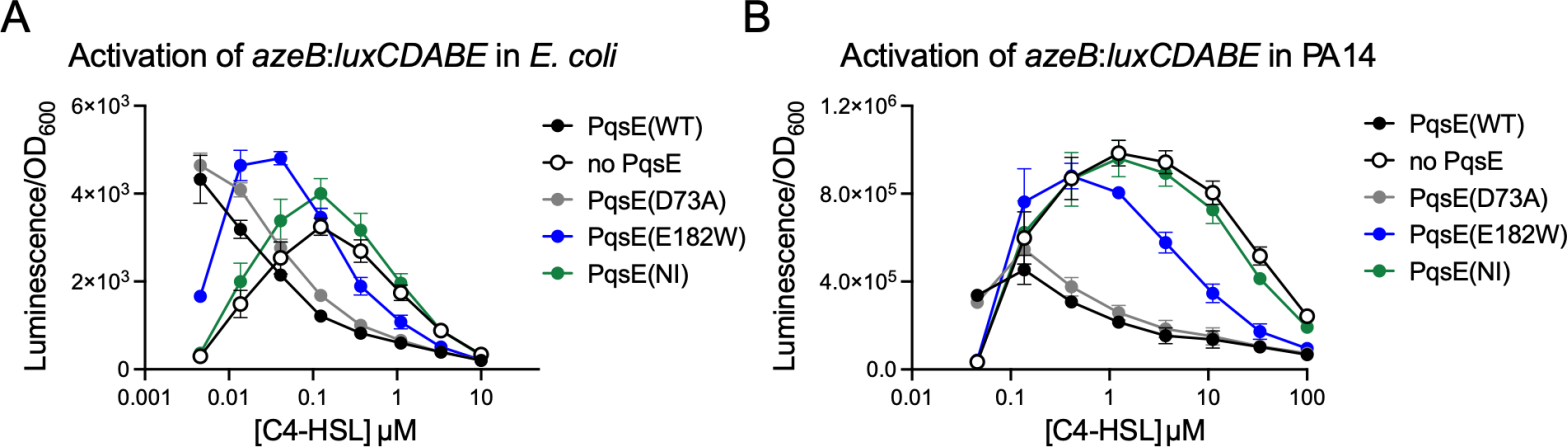
Activation of *azeB* by RhlR:C4-HSL is inhibited by PqsE binding. (**A**) Activation of *azeB-lux* in *E. coli* is reported as luminescence divided by OD_600_, and the measurements shown were collected after 17 h of growth. (**B**) Activation of *azeB-lux* in *P. aeruginosa* is reported as luminescence divided by OD_600_, and the measurements shown were collected after 17 h of growth. Results shown are the average of two independent experiments performed in duplicate. Error bars represent standard deviations.

In *E. coli*, we found that the dependence of RhlR on C4-HSL and PqsE binding followed a unique pattern that had not been observed in the heterologous model for any genes of interest previously (Fig. 4A). In the absence of C4-HSL, strains harboring an intact PqsE:RhlR complex [PqsE(WT) and PqsE(D73A)] produced greater signal than those in which the PqsE-RhlR interaction was abolished [no PqsE and PqsE(NI)], with the strain expressing PqsE(E182W) producing an intermediate level of luminescence. This trend became completely reversed with the addition of C4-HSL, where luminescence decreased in the strains expressing PqsE(WT) and PqsE(D73A) and increased in those lacking a stable interaction between PqsE and RhlR. Peak activity of RhlR was reached at ~40 nM C4-HSL in the strain expressing PqsE(E182W) and 100 nM in both the strain expressing no PqsE and the strain expressing PqsE(NI). High concentrations of C4-HSL decreased luminescence, bringing the signal down to baseline levels by 10 µM C4-HSL.

In *P. aeruginosa*, the activation of *azeB:luxCDABE* followed a similar trend to that observed in *E. coli*, at slightly altered concentration ranges of C4-HSL (Fig. 4B). As in *E. coli*, in the absence of C4-HSL, PqsE(WT) and PqsE(D73A) were both able to slightly boost activation of the transcriptional reporter. With the addition of mid-nanomolar concentrations of C4-HSL, the trend was reversed, showing that strains in which the PqsE-RhlR interaction was intact produced lower luminescence values. The signal reached peak levels at ~400 nM C4-HSL in the strain expressing PqsE(E182W) and ~1 µM C4-HSL in both the strains expressing no PqsE and PqsE(NI). Higher concentrations of C4-HSL decreased the activation of *azeB:luxCDABE* and reached near baseline levels at ~100 µM C4-HSL.

## DISCUSSION

**Fig 5:**
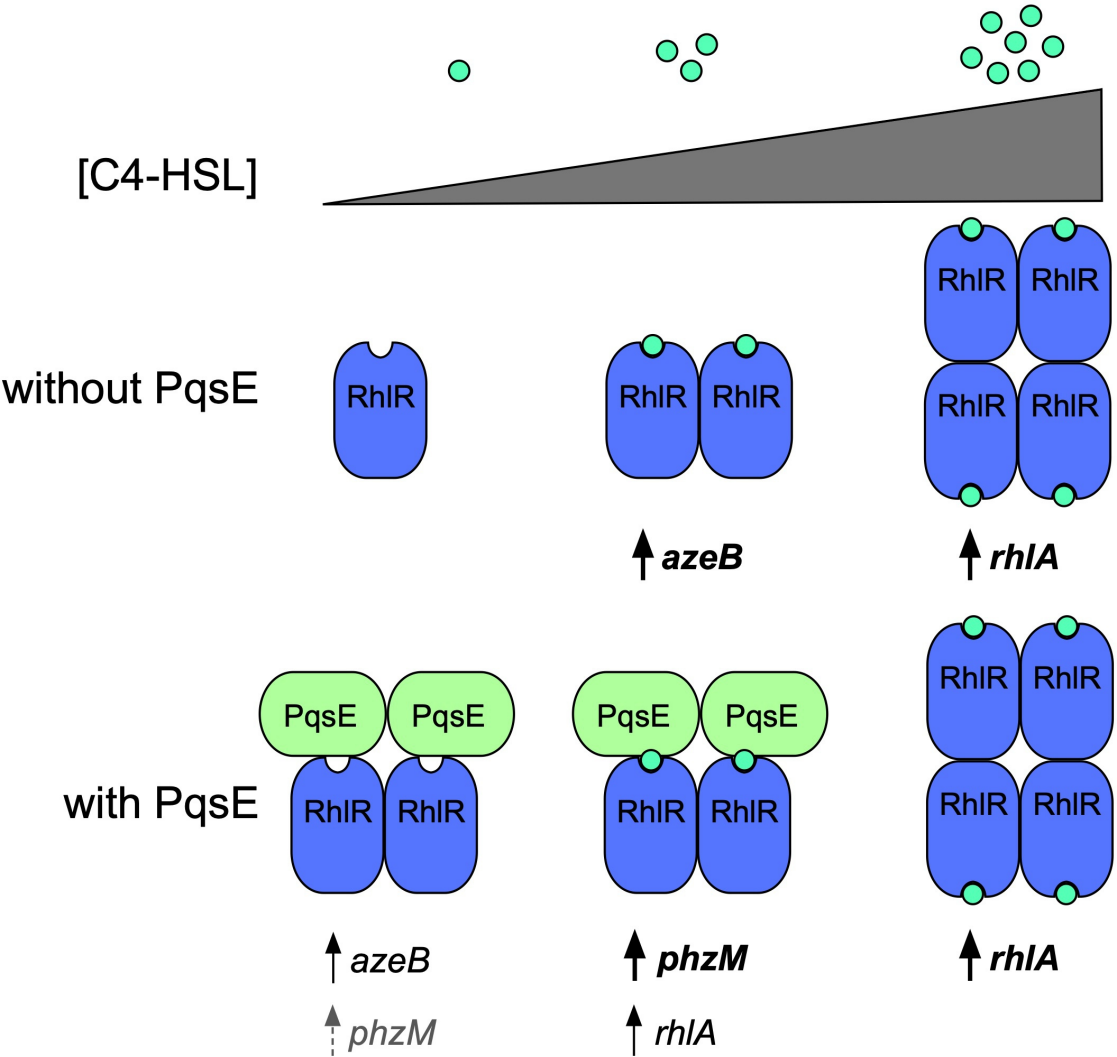
A proposed model for PqsE-adapted gene regulation by RhlR. At varying concentrations of C4-HSL, RhlR displays differential activation of the genes *rhlA*, *phzM*, and *azeB* in the presence and absence of an interaction with PqsE. The model reflects results obtained in this study that suggest increasing concentration of C4-HSL may induce multimerization of RhlR, whereas PqsE may act by stabilizing the dimeric species. Each species of RhlR is capable of activating subsets of genes in its regulon, except for the species that is not bound to C4-HSL or PqsE (here depicted as a monomer). Arrows and gene name fonts indicate the strength of activation, where dashed arrows with gray font indicate weak activation, solid arrows indicate intermediate activation, and bold arrows and font indicate the strongest level of activation observed for each gene. RhlR is in blue, PqsE is in green, and C4-HSL is represented as green circles.

In this study, we used a heterologous reporter system, along with a well-characterized set of PqsE variants with varying affinities for the transcription factor, RhlR, to precisely determine the contributions of each of RhlR’s binding partners, PqsE and C4-HSL, to the regulation of genes within the RhlR regulon. In each of the three cases explored here, expression patterns observed for RhlR target genes in the heterologous *E. coli* model closely resembled the patterns observed for these genes and their virulence factor products in *P. aeruginosa*. This finding suggests that there are no other regulatory factors in *P. aeruginosa* other than PqsE and C4-HSL that influence RhlR-dependent activation of the genes *rhlA*, *phzM*, and *azeB*. It was notable that, in the absence of an interaction with PqsE, high concentrations of C4-HSL had a stimulatory effect in the case of *rhlA* transcription but an inhibitory effect in the case of *azeB*. Because these expression patterns were observed in both *P. aeruginosa* and a non-native bacterium (*E. coli*), we suggest that this promoter-specific activity arises from the existence of multiple active conformations of RhlR, possibly involving multimeric species of the transcription factor (Fig. 5). Further work will be required to determine if this is the case and, if so, exactly what species of RhlR exist under what conditions. In Fig. 5, we present a model based on the findings presented herein.

The *P. aeruginosa* QS receptor/transcription factor, RhlR, is a unique LuxR family receptor. Not only does it rely on binding of its native ligand, C4-HSL, but it additionally requires an interaction with the protein, PqsE, for full functionality. Although eukaryotic transcription factors are known to rely on many complex protein-protein interactions (37), in the realm of prokaryotic gene regulation, this is unusual. It must be noted, however, that the binding of PqsE is not necessary for RhlR’s ability to activate transcription of some genes in its regulon (*rhlA* and *azeB* being the examples in this study). Rather, we hypothesize that RhlR may be able to adopt multiple conformational states, as stated above, and one of these states is stabilized by PqsE. The PqsE-stabilized state seems to be the only state capable of binding some promoters, such as for *phzM*, but not the only state capable of binding other promoters, like that of *rhlA*. It has been previously observed in PAO1 that certain genes in the RhlR regulon were downregulated as a result of increased PqsE expression (21). And in this study, it was noted that both high concentrations of C4-HSL and the presence of an intact PqsE-RhlR interaction inhibited the expression of *azeB*. While the previous study identified *azeB* (PA3327 in PAO1) as a PqsE-independent gene, it was not investigated for possible downregulation by PqsE. Genes that were shown to be downregulated by PqsE included *vqsR* and *clpP2* (21). It would be interesting to follow up on these genes and determine whether downregulation by PqsE is dependent on the interaction with RhlR or PqsE enzyme activity. Our use of the heterologous reporter reveals the complicated nature of the expression patterns observed for *azeB*. It appears PqsE binding does activate RhlR in the absence of C4-HSL, but even at low nanomolar concentrations of C4-HSL, PqsE binding switched to decrease activation of this gene.

Taken together, we propose that PqsE is acting as an adaptor protein for RhlR, adjusting its transcription factor activity in a promoter-specific manner and in response to varying concentrations of C4-HSL. While the terminology regarding classes of interacting partners for eukaryotic transcription factors is unclear and often inconsistent (they can be called adaptors, accessory proteins, co-activators, etc.) (37–39), we refer to PqsE as an adaptor protein due to its ability to adapt the activity of RhlR both to the identity of the promoter it is binding to and external conditions such as cell density. The interaction with PqsE seemingly affords RhlR activity that is tuned to produce different C4-HSL concentration-dependent expression patterns for subsets of genes in its regulon (Fig. 5). Perhaps there are conditions, such as the presence of certain metabolites, that could influence the formation of the PqsE-RhlR complex. While the three genes studied herein did not exhibit differential regulation between the *E. coli* and *P. aeruginosa* models, there could be other genes within the RhlR regulon that are controlled by additional factors in *P. aeruginosa*. The alignment of results between species could also depend on the culture conditions applied in this study, and we are currently exploring how varying growth modes could additionally influence genetic regulation in this system.

It is easy to imagine why *P. aeruginosa* would have evolved such a mechanism to fine-tune the regulation of genes responsible for the production of its various virulence factors. Through QS, *P. aeruginosa* senses the extracellular concentration of C4-HSL in order to gauge the number of neighboring *P. aeruginosa* cells in its vicinity. The production of rhamnolipids confers several benefits to *P. aeruginosa* in addition to toxicity against other species of bacteria, including biofilm formation, swarming motility, and nutrient acquisition (40). There is no drawback to the production of rhamnolipids when *P. aeruginosa* is at high cell density. Pyocyanin acts as a toxin against other competing species of bacteria as well as host cells. However, pyocyanin is also toxic to *P. aeruginosa* itself (41, 42). The expression of specialized enzymes that decompose the molecule serves as a mechanism to partially protect *P. aeruginosa* from self-toxicity of pyocyanin (42). This protective mechanism is limited, and therefore, it is logical that, when exposed to high concentrations of C4-HSL (signifying many neighboring *P. aeruginosa* cells), *P. aeruginosa* should stop expressing the genes involved in pyocyanin production. Recently, it was shown that an additional interaction between PqsE and the phosphodiesterase, ProE, also influences pyocyanin production, although how this interaction could potentially influence other QS behaviors is still unclear (43). It is intriguing that PqsE inhibits RhlR-activated transcription of some genes, such as *azeB*. It begs the question of what unintended effects might arise from the inhibition of the PqsE-RhlR interaction, as this is currently being explored as an antibiotic route. More work must be done to characterize the roles of molecules produced by the *aze* biosynthetic pathway. Regardless, the tunability of the PqsE-RhlR system serves as an example of the heightened adaptability of *P. aeruginosa*, and most likely contributes to the pathogenicity of this notorious bacterium.

## MATERIALS AND METHODS

### Strains, media, and molecular procedures

All *P. aeruginosa* strains were generated in the UCBPP-PA14 parental strain background, and all *E. coli* reporter strains were generated in the Top10 parental strain background. Unless otherwise stated, all strains were grown in Luria-Bertani (LB) broth (Difco DF0446) and supplemented with appropriate selective antibiotics at the following concentrations: carbenicillin (400 µg/mL), ampicillin (200 µg/mL), kanamycin (100 µg/mL), and chloramphenicol (10 µg/mL). Plasmids were transformed into *P. aeruginosa* strains as described (44) and into *E. coli* strains by electroporation. A list of all strains featured in this study is included in the Supporting Information (Table S1).

### Generation of *azeB-lux* reporters

Both the pCS26-*azeB-luxCDABE* plasmid (*E. coli* reporter) and a pUC18T-mini-Tn7T-P*azeB-lux*-Tp plasmid for the construction of a *P. aeruginosa azeB* reporter were assembled following a previously established protocol (45). Briefly, the 470-bp region upstream of *azeB* and each vector were amplified to introduce ~20 bp overlaps. The amplified vectors were digested with Dpn1, and 1 µL of each (vector and promoter region insert) was transformed into ultracompetent XL-10 Gold cells (Agilent) for *in vivo* assembly. Positive clones were confirmed by sequencing, and the isolated pCS26-*azeB-luxCDABE* plasmid was transformed into *E. coli* Top10 to generate the heterologous reporter. The pUC18T-mini-Tn7T-P*azeB-lux*-Tp plasmid was transformed into Top10 and mated with *P. aeruginosa* PA14 to stably integrate the *azeB-luxCDABE* fusion at the att-Tn7 site next to *glmS*.

### Heterologous reporters of RhlR transcription factor activity

*E. coli* strains harboring plasmids with *rhlR* driven by the P_BAD_ promoter, *luxCDABE* under the P*rhlA*, P*phzM*, or P*azeB* promoter on the pCS26 vector, and either the pACYC184 vector or pACYC184 harboring WT *pqsE* or mutant *pqsE* alleles were grown with shaking overnight at 37°C in LB medium supplemented with ampicillin, kanamycin, and chloramphenicol. Overnight cultures were diluted 1:1,000 into fresh LB with antibiotics, 0.1% arabinose, and varying concentrations of C4-HSL were then added to the wells of a white, clear-bottomed 96-well plate (Corning 3903) to a total volume of 100 µL per well. Plates were incubated in the plate-reader (Molecular Devices iD5) at 30°C with periodic shaking for 24 h, and luminescence and OD_600_ were measured every 10 minutes. Bioluminescence values are reported as the luminescence measurement divided by OD_600_, and the timepoints shown are the time at which peak signal was observed (typically at 17 h).

### *rhlA:mNeonGreen* assay

*∆rhlI ∆pqsE P. aeruginosa* PA14 strains harboring *mNeonGreen* under the *rhlA* promoter and either a pUCP18 control vector or pUCP18 harboring WT *pqsE* or mutant *pqsE* alleles were grown with shaking overnight at 37°C in LB medium supplemented with carbenicillin. Overnight cultures were diluted 1:1,000 into fresh LB with carbenicillin, and varying concentrations of C4-HSL were then added to the wells of a round-bottomed 96-well plate (Corning 3797) to a total volume of 100 µL per well. The lid and a Breathe-EASIER plate sealing film (Diversified Biotech BERM-2000) were applied to minimize evaporation during shaking overnight at 37°C. The following day, cells were pelleted by centrifugation at 2,000 rpm for 20 minutes at 4°C. The supernatant was removed, and cell pellets were resuspended in 100 µL of PBS (137 mM NaCl, 2.7 mM KCl, 10 mM Na_2_HPO_4_ 1.8 mM KH_2_PO_4_, pH 7.4), per well. Cell suspensions were transferred into the wells of a black, clear-bottomed 96-well plate (Corning 3904), and fluorescence (excitation at 485 nm; emission at 535 nm) and OD_600_ were measured in a plate-reader (Molecular Devices iD5).

### Pyocyanin assay

Overnight cultures of ∆*rhlI P. aeruginosa* strains harboring chromosomally encoded *pqsE* alleles (or the *pqsE* deletion) were grown from single colonies in LB medium at 37°C. Cultures were diluted 1:1,000 in 2 mL of fresh LB liquid medium supplemented with C4-HSL (1% DMSO) at the specified concentrations and grown with shaking at 37°C for 17 h. Cells were then pelleted by centrifugation, and OD_695_ of the clarified supernatants was measured in a Genesys 20 ThermoSpectronic spectrophotometer. Pellets were resuspended in PBS, and OD_600_ was measured to determine cell density of each sample. Pyocyanin production is reported as the OD_695_ of clarified supernatant normalized to the OD_600_ of the resuspended pellet.

### *P. aeruginosa azeB:lux* assay

*∆rhlI ∆pqsE P. aeruginosa* PA14 strains harboring *luxCDABE* under the P*azeB* promoter and either a control pUCP18 vector or pUCP18 harboring WT *pqsE* or mutant *pqsE* alleles were grown with shaking overnight at 37°C in LB medium supplemented with carbenicillin. Overnight cultures were diluted 1:1,000 into fresh LB with carbenicillin, and varying concentrations of C4-HSL were then added to the wells of a white, clear-bottomed 96-well plate (Corning 3903) to a total volume of 100 µL per well. Plates were incubated in the plate-reader (Molecular Devices iD5) at 30°C with periodic shaking for 17 h, and luminescence and OD_600_ were measured every 10 minutes.

## SUPPLEMENTAL MATERIAL

Table S1 jb.00516-24-s0001.pdf All strains included in study.

ASM does not own the copyrights to Supplemental Material that may be linked to, or accessed through, an article. The authors have granted ASM a non-exclusive, world-wide license to publish the Supplemental Material files. Please contact the corresponding author directly for reuse.
