# Supplementary material for: PqsE adapts the activity of the Pseudomonas aeruginosa quorum-sensing transcription factor RhlR to both autoinducer concentration and promoter sequence identity
Source: J Bacteriol. 2025 Apr 17;207(5):e00516-24. doi: 10.1128/jb.00516-24 (PMC12096825; doi:10.1128/jb.00516-24)
Supplement: Table S1 — All strains included in study. [file jb.00516-24-s0001.pdf]

Supporting Information for

PqsE adapts activity of the *Pseudomonas aeruginosa* quorum-sensing transcription factor RhlR to both autoinducer concentration and promoter sequence identity

Bilalay V. Tchadi[1], Jesse J. Derringer[1], Anna K. Detweiler[1], Isabelle R. Taylor[1#]

[1]Department of Chemistry, William & Mary, Williamsburg, VA 23185, USA

[#]To whom correspondence should be addressed. Email: irtaylor@wm.edu

This PDF file includes:

Table S1

Supplementary Tables

Strain	Description	Reference
UCBPP-PA14	PA14 *P. aeruginosa* Wildtype	Laboratory stock
SM1328	*E. coli* Top10 *P_{BAD}-rhlR PrhlA-luxCDABE pACYC184(control)*	14
IT116	*E. coli* Top10 *P_{BAD}-rhlR PrhlA-luxCDABE pACYC184_DP-P_{lac}-pqsE(WT)*	17
IT117	*E. coli* Top10 *P_{BAD}-rhlR PrhlA-luxCDABE pACYC184_DP-P_{lac}-pqsE(D73A)*	17
IT118	*E. coli* Top10 *P_{BAD}-rhlR PrhlA-luxCDABE pACYC184_DP-P_{lac}-pqsE(E182W)*	17
IT119	*E. coli* Top10 *P_{BAD}-rhlR PrhlA-luxCDABE pACYC184_DP-P_{lac}-pqsE(NI)*	17
JD17	PA14 *ΔrhlIΔpqsE PrhlA-mNeonGreen pUCP18-P_{lac}-pqsE(WT)*	This study
JD18	PA14 *ΔrhlIΔpqsE PrhlA-mNeonGreen pUCP18(control)*	This study
JD19	PA14 *ΔrhlIΔpqsE PrhlA-mNeonGreen pUCP18-P_{lac}-pqsE(D73A)*	This study
JD20	PA14 *ΔrhlIΔpqsE PrhlA-mNeonGreen pUCP18-P_{lac}-pqsE(E182W)*	This study
JD21	PA14 *ΔrhlIΔpqsE PrhlA-mNeonGreen pUCP18-P_{lac}-pqsE(NI)*	This study
IT172	*E. coli* Top10 *P_{BAD}-rhlR PphzM-luxCDABE pACYC184(control)*	This study
IT173	*E. coli* Top10 *P_{BAD}-rhlR PphzM-luxCDABE pACYC184_DP-P_{lac}-pqsE(WT)*	This study
IT174	*E. coli* Top10 *P_{BAD}-rhlR PphzM-luxCDABE pACYC184_DP-P_{lac}-pqsE(D73A)*	This study
IT175	*E. coli* Top10 *P_{BAD}-rhlR PphzM-luxCDABE pACYC184_DP-P_{lac}-pqsE(E182W)*	This study
IT176	*E. coli* Top10 *P_{BAD}-rhlR PphzM-luxCDABE pACYC184_DP-P_{lac}-pqsE(NI)*	This study
SM52	PA14 Δ*rhlI*	17
IT122	PA14 Δ*rhlI* ΔpqsE	17
IT123	PA14 Δ*rhlI pqsE(D73A)*	17
IT124	PA14 Δ*rhlI pqsE(E182W)*	17
IT129	PA14 Δ*rhlI pqsE(NI)*	17
BT013	PA14 *ΔrhlIΔpqsE PazeB-luxCDABE pUCP18-P_{lac}-pqsE(WT)*	This study
BT014	PA14 *ΔrhlIΔpqsE PazeB-luxCDABE pUCP18(control)*	This study
BT015	PA14 *ΔrhlIΔpqsE PazeB-luxCDABE pUCP18-P_{lac}-pqsE(D73A)*	This study
BT016	PA14 *ΔrhlIΔpqsE PazeB-luxCDABE pUCP18-P_{lac}-pqsE(E182W)*	This study
BT017	PA14 *ΔrhlIΔpqsE PazeB-luxCDABE pUCP18-P_{lac}-pqsE(NI)*	This study
BT029	*E. coli* Top10 *P_{BAD}-rhlR PazeB-luxCDABE pACYC184_DP-P_{lac}-pqsE(WT)*	This study
BT030	*E. coli* Top10 *P_{BAD}-rhlR PazeB-luxCDABE pACYC184(control)*	This study
BT031	*E. coli* Top10 *P_{BAD}-rhlR PazeB-luxCDABE pACYC184_DP-P_{lac}-pqsE(D73A)*	This study
BT032	*E. coli* Top10 *P_{BAD}-rhlR PazeB-luxCDABE pACYC184_DP-P_{lac}-pqsE(E182W)*	This study
BT033	*E. coli* Top10 *P_{BAD}-rhlR PazeB-luxCDABE pACYC184_DP-P_{lac}-pqsE(NI)*	This study

Table S1. Strains used in this study.
